# Supplementary material for: Discovery and application of insertion-deletion (INDEL) polymorphisms for QTL mapping of early life-history traits in Atlantic salmon
Source: BMC Genomics. 2010 Mar 8;11:156. doi: 10.1186/1471-2164-11-156 (PMC2838853; doi:10.1186/1471-2164-11-156)
Supplement: Additional file 2 — Information on developed 76 locus single-run INDEL panel in Atlantic salmon. Information on fluorescence labeling, primer concentrations, PCR pooling and links to alignments, INDEL motifs and GENESCAN (Burge and Karlin 1997) predictions of genes/exons are available in html format. [file 1471-2164-11-156-S2.ZIP › Additionalfile2/snpsummary8417.html]

```
Cluster 2629 Contig 2

prev  Summary    Contig List  next
```

Size of Consensus sequence = 725

Number of sequences = 8

Minimum redundancy = 3

Key

A gi|117832711|gb|EG905407.1|EG905407 EST\_ssal\_evf\_8023 ssalevf mixed\_tissue Salmo salar cDNA Salmo salar cDNA clone ssal\_evf\_509\_028\_fwd 3', mRNA sequence  
B gi|117832712|gb|EG905408.1|EG905408 EST\_ssal\_evf\_8024 ssalevf mixed\_tissue Salmo salar cDNA Salmo salar cDNA clone ssal\_evf\_509\_028\_rev 5', mRNA sequence  
C gi|117441285|gb|EG773508.1|EG773508 EST\_ssal\_evd\_48942 ssalevd thymus Salmo salar cDNA Salmo salar cDNA clone ssal\_evd\_565\_312\_rev 5', mRNA sequence  
D gi|117450100|gb|EG782319.1|EG782319 EST\_ssal\_evd\_38872 ssalevd thymus Salmo salar cDNA Salmo salar cDNA clone ssal\_evd\_551\_355\_fwd 3', mRNA sequence  
E gi|117476133|gb|EG808352.1|EG808352 EST\_ssal\_evd\_28836 ssalevd thymus Salmo salar cDNA Salmo salar cDNA clone ssal\_evd\_537\_298\_rev 5', mRNA sequence  
F gi|117450101|gb|EG782320.1|EG782320 EST\_ssal\_evd\_38873 ssalevd thymus Salmo salar cDNA Salmo salar cDNA clone ssal\_evd\_551\_355\_rev 5', mRNA sequence  
G gi|117528381|gb|EG860022.1|EG860022 EST\_ssal\_eve\_53311 ssaleve thyroid Salmo salar cDNA Salmo salar cDNA clone ssal\_eve\_572\_249\_rev 5', mRNA sequence  
H gi|117459885|gb|EG792104.1|EG792104 EST\_ssal\_evd\_12213 ssalevd thymus Salmo salar cDNA Salmo salar cDNA clone ssal\_evd\_515\_075\_rev 5', mRNA sequence

3 SNPs detected

A B C D E F G H  cosegregation weighted

224 - - - T T T . T   3/3 87.50
225 - - - A A A . A   3/3 87.50
389 A A A C C C A C   3/3 100.00
